# Whole-genome sequencing reveals transmission pattern and drug resistance of *Mycobacterium tuberculosis* intra- or inter-hosts

**DOI:** 10.3389/fcimb.2024.1488547

**Published:** 2025-01-21

**Authors:** Feng Ding, Wanfei Liu, Chi Wu, Wensi Zhang, Shuyan Chen, Wenjie Lai, Jiayao Qu, Qiang Lin, Shuihua Lu, Jiuxin Qu

**Affiliations:** ^1^ National Clinical Research Center for Infectious Diseases, Shenzhen Third People’s Hospital, Shenzhen, China; ^2^ Shenzhen Branch, Guangdong Laboratory for Lingnan Modern Agriculture, Genome Analysis Laboratory of the Ministry of Agriculture and Rural Affairs, Agricultural Genomics Institute at Shenzhen, Chinese Academy of Agricultural Sciences, Shenzhen, China; ^3^ Department of Clinical Laboratory, Shenzhen Third People’s Hospital, The Second Affiliated Hospital of Southern University of Science and Technology, Shenzhen, China

**Keywords:** *Mycobacterium tuberculosis*, transmission pattern, whole genome sequencing, phenotypic DST, drug resistant mutations

## Abstract

**Background:**

Tuberculosis (TB) remains a serious global public health problem. The *Mycobacterium tuberculosis* (MTB) is responsible for approximately 10 million new TB cases globally each year. This study aimed to investigate transmission pattern and drug resistance of MTB in Shenzhen, China.

**Methods:**

A retrospective study on 286 samples from 184 TB patients collected between 2015 and 2018 in Shenzhen Third People’s Hospital was conducted using whole-genome sequencing. Drug susceptibility testing (DST) was performed using both phenotypic DST (pDST) and molecular DST (mDST). Sample diversity was evaluated by SNPs and transmission clusters were identified based on SNP differences of 12 or fewer in genetic clusters.

**Results:**

Except four samples identified as non-tuberculous mycobacteria, 282 MTB samples (181 patients) underwent mDST, with 244 samples (162 patients) undergoing pDST. The overall multidrug-resistant rate in patients was 22.31% in pDST (12.00% for new patients and 40.82% for retreatment patients) and 34.48% in mDST (20.41% for new patients and 58.21% for retreatment patients). Totally 92 transmission clusters were identified, encompassing 70.21% samples (57.46% patients), with 5 clusters containing samples (15, 5.32%) from different patients (9, 4.97%), indicating recent transmission. The drug-resistant mutations in 36 of 45 transmission clusters (80.00%) were identical in all samples, suggesting the transmission of drug resistance. Patients with multiple samples were categorized into simultaneous sampling (SS) and continuous sampling (CS) groups, revealing significant differences in treatment types, treatment outcomes, residential addresses, and drug resistance types. mDST showed greater accuracy than pDST in SS and CS groups. A novel method based on heterozygous SNPs and two-sample Kolmogorov–Smirnov test were developed and identified 12 (4.26%) samples as mixed infection samples. Six of 12 patients had mixed and pure samples together, and major strains of mixed samples were closer to corresponding pure strains than minor strains.

**Conclusions:**

This retrospective study, conducted at the only municipal hospital specializing in infectious diseases in Shenzhen, provides the opportunity to understand drug resistance of TB patients, which mainly are refractory patients. The study revealed transmission patterns of MTB, analyzed mixed infections, and tracked changes in MTB strains during short/long-term treatment.

## Introduction

Tuberculosis (TB) remains a serious global public health problem. Approximately 15% of the TB cases exhibit resistance to rifampicin (RFP) or isoniazid (INH) (WHO, 2020). Whole-genome sequencing (WGS) provides an ultimate resolution for *Mycobacterium tuberculosis* (MTB) study, which can be used for MTB diagnosis (Coll et al., 2014), drug susceptibility profiling (CRyPTIC Consortium, the 100,000 Genomes Project, 2018), and understanding of the transmission of MTB (Nikolayevskyy et al., 2019). MTB lineages can be identified based on phylogenetically robust markers such as single-nucleotide polymorphisms (SNPs) or large sequence polymorphisms (Comas et al., 2009; Coll et al., 2014). Molecular drug susceptibility testing (mDST) by WGS is also compatible with phenotypic drug sensitivity testing (pDST) for fist-line drugs and several second-line drugs (Bradley et al., 2015; CRyPTIC Consortium, the 100,000 Genomes Project, 2018). Based on the mutation rate and epidemiological observations, a cutoff value of fewer than six SNPs has been proposed to indicate recent transmission, which can be used for identification of isolates involved in direct human-to-human TB transmission (Nikolayevskyy et al., 2016; Nikolayevskyy et al., 2019).

Analysis of emergence, spread, and drug resistance of MTB isolates in a specific geographical area can facilitate disease control and treatment. However, transmission patterns of TB in most areas of China are still not clear (Yang and Gao, 2018). The transmission of multidrug-resistant (MDR) TB was 5% between 2009 and 2012 in Shanghai (Yang et al., 2017). In Shenzhen, previous studies showed that the overall MDR rate was 4.20% during 2000–2013 (Zhu et al., 2017), 5.08% during 2013–2017 (Jiang et al., 2020), and 6.66% during 2014–2017 (Yang et al., 2021). To further evaluate the transmission and drug resistance of MTB, a retrospective study was conducted on isolates collected in Shenzhen Third People’s Hospital, China, between 2015 and 2018.

We collected 500 culture-positive samples from 265 TB patients, performed WGS on 286 samples from 184 patients, and conducted pDST on 244 samples from 162 patients. Using WGS data, we analyzed lineage, drug resistance, and transmission pattern for all 286 samples. Moreover, we also evaluated the polyclonal infection and change of MTB in long-term treatment.

## Methods

### Study population

During 2015–2018, a total of 500 culture-positive samples from 265 TB patients were collected at Shenzhen Third People’s Hospital, which is the only municipal hospital specializing in infectious diseases in Shenzhen. Most patients were refractory cases referred from district-level infectious disease hospitals across Shenzhen. Among them, 210 patients had multiple samples and 55 patients had a single sample. Sample types included sputum (244, 48.80%), bronchoalveolar lavage fluid (103, 20.60%), pleural fluid (82, 16.40%), tissue (16, 3.20%), cerebrospinal fluid (12, 2.40%), pus (12, 2.40%), urine (11, 2.20%), and others (ascitic fluid, feces, secretion, drainage fluid, blood, puncture fluid, semen, and urine).

All samples underwent WGS using the Illumina HiSeq 2000 platform (Illumina, San Diego, CA), resulting in high-quality WGS data for 286 samples from 184 patients. Samples with high-quality sequencing data were further analyzed for pDST by determining minimal inhibitory drug concentrations using TREK Sensititre MYCOTB plates (TREK Diagnostic Systems, Oakwood, OH) for 12 drugs (Lee et al., 2014). To investigate the impact of sampling intervals on samples from the same patient, patients with multiple samples were categorized into two groups: simultaneous sampling (SS) patients with a sampling intervals of less than 2 months and continuous sampling (CS) patients with a sampling intervals of more than 2 months. WGS analysis yielded data for 55 SS patients (114 samples) and 21 CS patients (45 samples). Clinical data were obtained according to the patient’s electronic medical record.

### Whole-genome sequencing

Sample was re-cultured, and DNA was extracted by the CTAB method (Somerville et al., 2005). A paired-end library was constructed, and WGS was carried out. Finally, 150-bp paired-end reads were produced for each sample with an average coverage of 264.77X. The raw sequence data were deposited in the Genome Sequence Archive at the National Genomics Data Center, Beijing Institute of Genomics, Chinese Academy of Sciences/China National Center for Bioinformation, which can be accessed at https://bigd.big.ac.cn/gsa (GSA: CRA018968) (Chen et al., 2021; CNCB-NGDC Members and Partners, 2021). After sequencing, adapter and low-quality sequence of raw data were filtered by Trimmomatic (version 0.39) (Bolger et al., 2014). The low-quality bases below quality 20 were removed, and the reads less than 40 bases were filtered. Filtered reads were aligned to *M. tuberculosis* H37Rv genome (NC_000962) using BWA software (BWA-mem, version 0.75a-r405) (Li and Durbin, 2009). SNPs were identified by the Genome Analysis Toolkit (GATK, version 4.1.4.1) (DePristo et al., 2011). SNPs with read depth <3 or allelic depth <3 were removed, and the remain SNPs were used for subsequent analysis. The phylogenetic lineage was identified by KvarQ (version 0.12.3a1) (Steiner et al., 2014).

### Species determination

To identify species, the specific markers of species were identified by comparing the rRNA genes (*rrs* and *rrl*) of *M. tuberculosis* H37Rv with other species in the Mycobacteriaceae family (totally 508 strains of 198 species with refseq bacteria assembly) (O'Leary et al., 2016). Totally 2,065 SNP markers were used for species determination, and the accuracies of species and group [MTB and non-tuberculous mycobacteria (NTM)] prediction for 508 reference strains were 99.61% (506 of 508) and 100%, respectively. By comparing sequence variant files of each sample with these markers using an in-house Perl script, the species of sample was identified.

### Molecular drug susceptibility testing

SNPs with read depth <3 or allele depth <3 or allele frequency <0.1 were removed, and the remaining SNPs were used for molecular drug susceptibility testing (mDST) for 16 drugs by comparing them with known drug-resistant mutations (**supplementary Image 1**) (Qu et al., 2024). Types of drug resistance were obtained according to their definition: drug sensitivity (DS: susceptible to any antituberculous drugs), drug resistance (DR: resistant to any antituberculous drugs except for RFP), RFP resistance (RR: resistant to RFP), multidrug resistance (MDR: resistant to RFP and INH), preliminarily extensive drug resistance {Pre-XDR: fulfilled the definition of MDR/RR and resistant to any fluoroquinolone (FQ) [levofloxacin (LFX) or moxifloxacin (MFX)]}, and extensive drug resistance [XDR: fulfilled the definition of MDR/RR, resistant to any FQ (LFX or MFX), and resistant to at least one additional group A drug (bedaquiline or linezolid)] (WHO, 2021a). For convenience, drug resistance types are exclusive except for the MDR rate.

### Phylogeny and transmission analysis

Homozygous SNPs with variant allele frequency ≥95% in the gene region (excluding 163 PE/PPE/PGRS genes and 53 drug-resistant genes) were extracted. Then, SNP loci with missing genotype information exceeding 5% in the total samples were filtered out. After that, the remaining SNPs were used to construct concatenated alignment for all samples. The alignment was used to generate a neighbor-joining phylogenetic tree by Clustalw2 (version 2.0.12) (Larkin et al., 2007) and a maximum likelihood phylogenetic tree using PhyML (version 3.3.20190909) (Guindon et al., 2010). The phylogenetic tree was drawn using EvolView3 (Subramanian et al., 2019). The common SNPs for any node in the phylogenetic tree and the unique SNPs for each branch of the node were obtained by allele comparison. The population-based studies observed that 12 SNPs can be a potential threshold to define recent transmission in China (Yang et al., 2017) and worldwide (Walker et al., 2013). Therefore, the nodes with no more than 12 unique SNPs were assigned as transmission clusters in our study.

### Mixed infection analysis

Heterozygous SNPs with variant allele frequencies between 5% and 95% and sequence depth equal or more than 10 in gene regions were used for mixed infection analysis. To reduce the effect of genome regions with high variability, the inexact repeat regions were identified by MUMmer software (version 4.0.0rc1, nucmer for genome self-comparison, and mummer for gene–genome alignment) (Marçais et al., 2018). Genes overlapped with these inexact repeat regions were filtered. Furthermore, heterozygous SNPs located in rRNA, tRNA, ncRNA, PE/PPE/PGRS, transposase, and drug resistance genes were excluded.

In order to establish a reliable method for identification of mixed infection, a simulated mixed infection dataset was produced by DWGSIM software (version 0.1.11, with the following parameters: −e 0.0026, −E 0.0040, −d 300, −1 150, −2 150, and −r 0.000001) based on whole-genome sequences of six representative genomes for lineage 1 to lineage 6 (**Supplementary Table 2**) (Homer, 2022). The simulated data of each genome with different coverages (3X, 5X, 10X, 15X, 30X) were combined with other genomes (probability combination) to produce 100× mixed samples and totally 75 artificial mixtures were obtained. Genes with heterozygous SNPs in pure 100X simulated data of six representative genomes were identified as possible misalignment regions and excluded from mixed infection analysis. To further reduce the influence of heterozygous SNPs with extreme sequencing depth and allele frequency, only heterozygous SNPs within mean depth ± 3*standard deviation (SD) and mean allele frequency ± 3*SD (for major and minor alleles respectively) were used in downstream analysis. Based on simulated data, the allele frequency profiles of heterozygous SNPs showed a normal distribution, as shown in a previous study (Sobkowiak et al., 2018). According to this, we constructed the reference allele frequency profiles of heterozygous SNPs in the mixtures above (3X + 97X, 5X + 95X, 10X + 90X, 15X + 85X, 30X + 70X) and imputed the reference allele frequency profiles for other possible mixtures by linear interpolation. Compared with the reference allele frequency profiles, 68 of 75 (90.67%) artificial mixtures were identified as mixed infection by two-sample Kolmogorov–Smirnov test (P value is equal to or larger than 0.01) (Marsaglia et al., 2003). Among seven false negative mixtures, one mixture only had 3X minority strain and the other six mixtures had 5X minority strains. To filter possible false positive identification, we added other criteria as follows: a) the number of heterozygous SNPs after being filtered by depth and allele frequency in majority or minority strains ≥6; b) the total number of heterozygous SNPs ≥12; and c) the proportion of heterozygous SNPs to total SNPs ≥0.015 when heterozygous SNPs are lower than 18, or ≥0.02 when heterozygous SNPs are equal to or larger than 18. The new criteria did not affect the identification of mixed infection for all 75 simulated mixtures.

To estimate method performance, an artificial mixed dataset was created based on eight clinical sequencing data in our study (two samples from each lineage of lineage 1, lineage 2, lineage Beijing, and lineage 4). Sequence data of each sample with different coverages (3X, 5X, 10X, 15X, 30X) were extracted using seqtk software (version: 1.3-r117-dirty) and combined with other samples (within and between lineages) to generate 100X mixtures (**Supplementary Table 3**) (Li, 2012). Thus, totally 140 100X mixtures (20 within lineages and 120 between lineages) were obtained. Compared with reference allele frequency profiles, 132 of 140 (94.29%) artificial mixtures were identified as mixed infection. Among eight false negative mixtures, six mixtures only had 3X minority strain, one mixture had 5X minority strain, and one mixture had 10X minority strain. Finally, mixed infection of each sample in our study was predicted based on this method.

## Results

### Baseline characteristics of the cohort

**Figure 1:**
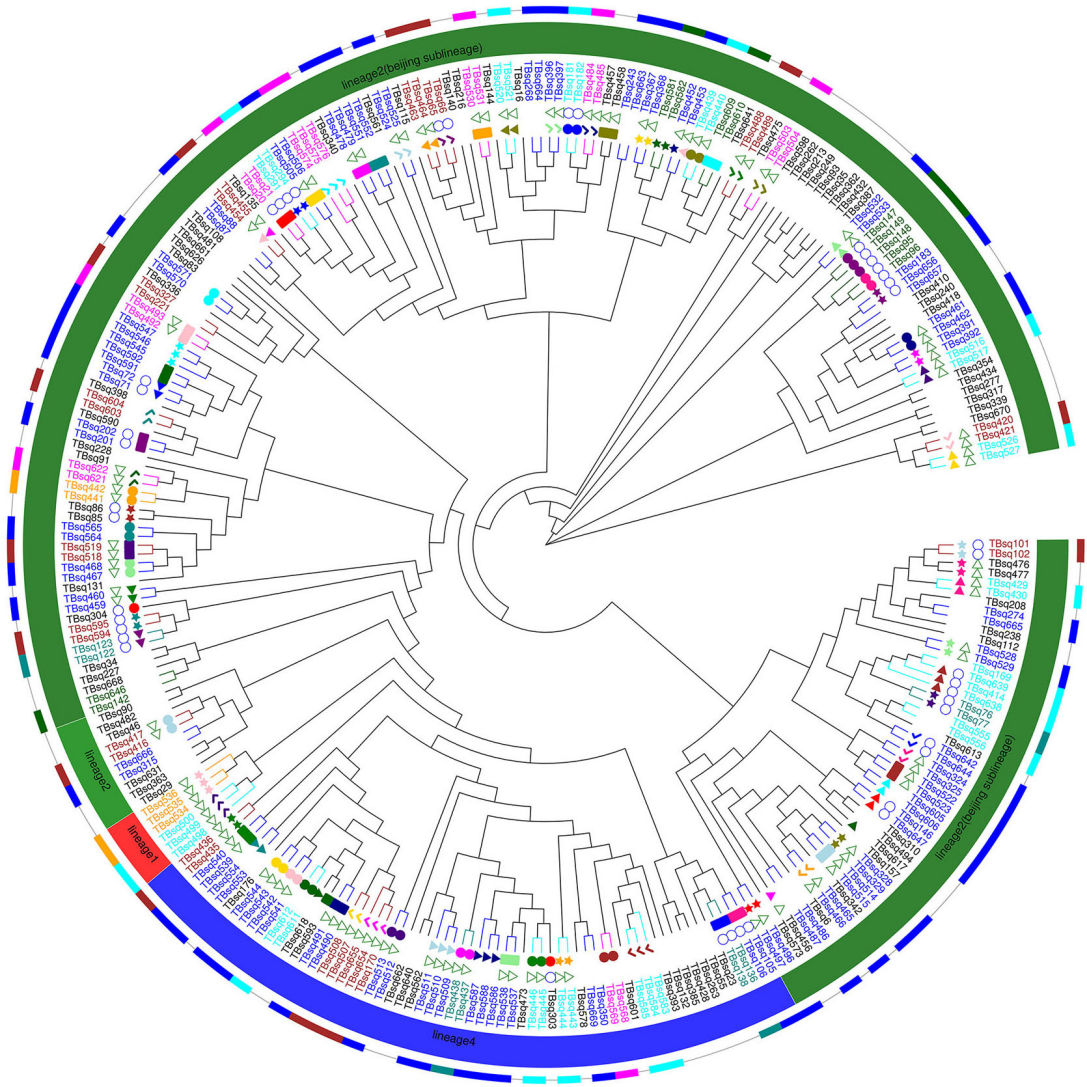
The phylogenetic tree of 282 MTB samples. The different colors on branches and leaf labels indicate different specific SNP numbers in transmission clusters (blue: 0; brown: 1; cyan: 2; dark cyan: 3; dark green: 4; magenta: 5; and orange: 6). Two columns of leaf decorations are shown for patients with more than one sample (unique solid color shape for each patient) and patients in simultaneous sampling (open green triangle) and continuous sampling (open blue circle) categories. Sample lineage is shown next with a leaf label with a background color (lineage 1: red; lineage 2: green; Beijing sublineage (lineage 2): dark green; and lineage 4: blue) and transmission clusters are shown with column plot (colored as leaf labels).

A total of 500 culture-positive samples from 265 TB patients were collected in Shenzhen Third People’s Hospital, China, between 2015 and 2018 (**Supplementary Figure 1**). Among these patients, 71.70% (190/265) were men, 55.47% (147/265) were newly diagnosed patients, 52.45% (139/265) had improved treatment outcomes, and the median age was 40 years (interquartile range, 30–56) (**Table 1**). Sequencing data were obtained for 286 samples from 184 patients. Compared with non-sequenced patients (sequencing failed), successfully sequenced patients had more samples and better treatment outcomes, although the differences were not significant (χ2 = 7.6128, P = 0.0547 for samples and χ2 = 8.6555, P = 0.0703 for treatment outcomes). Four (1.40%, belonging to four patients) of 286 samples were identified as NTM by comparing with species markers in rRNA genes (*rrs* and *rrl*) of the Mycobacteriaceae family (one *Mycobacteroides abscessus*, one *Mycobacterium gordonae*, one *Mycobacterium intracellulare*, and one *Mycobacterium kansasii*). All these NTM species were among the most common clinical NTM species in Guangdong province, China (Zhou et al., 2020). The homozygous SNPs in 282 MTB samples were used for sequence alignment and phylogeny tree construction using the neighbor-joining method (**Figure 1**; **Supplementary Table 4**). Among these samples, 2.13% (6/282) belonged to lineage 1, 75.53% (213/282) belonged to lineage 2, 71.99% (203/282) belonged to Beijing sublineage), and 22.34% (63/282) belonged to lineage 4.

**Table 1:** The clinical characteristics of TB patients.

Data	Number			X-squared	P value
	Total	Sequenced	Non-sequenced		
Gender	265	184	81	0.0290	0.8647
Male	190 (71.70%)	133 (72.28%)	57 (70.37%)		
Female	75 (28.30%)	51 (27.72%)	24 (29.63%)		
Patient samples	265	184	81	**7.6128**	**0.0547**
1 sample	55 (20.75%)	30 (16.30%)	25 (30.86%)		
2 samples	188 (70.94%)	137 (74.46%)	51 (62.96%)		
3 samples	19 (7.17%)	15 (8.15%)	4 (4.94%)		
4 samples	3 (1.13%)	2 (1.09%)	1 (1.23%)		
Age	265	184	81	3.6874	0.5952
≤20	13 (4.91%)	11 (5.98%)	2 (2.47%)		
21-30	64 (24.15%)	46 (25.00%)	18 (22.22%)		
31-40	57 (21.51%)	36 (19.57%)	21 (25.93%)		
41-50	48 (18.11%)	33 (17.93%)	15 (18.52%)		
51-60	30 (11.32%)	23 (12.50%)	7 (8.64%)		
>60	53 (20.00%)	35 (19.02%)	18 (22.22%)		
Treatment	265	184	81	3.7242	0.1553
New	147 (55.47%)	106 (57.61%)	41 (50.62%)		
Retreatment	95 (35.85%)	66 (35.87%)	29 (35.80%)		
Unknown	23 (8.68%)	12 (6.52%)	11 (13.58%)		
**Treatment outcome**	265	184	81	**8.6555**	**0.0703**
Improved	139 (52.45%)	102 (55.43%)	37 (45.68%)		
Stable	50 (18.87%)	35 (19.02%)	15 (18.52%)		
Worse*	7 (2.64%)	6 (3.26%)	1 (1.23%)		
Under treatment^#^	62 (23.40%)	39 (21.20%)	23 (28.40%)		
Unknown	7 (2.64%)	2 (1.09%)	5 (6.17%)		
Residential address	265	184	81	4.1625	0.1248
Non-Shenzhen	15 (5.66%)	13 (7.07%)	2 (2.47%)		
Shenzhen	182 (68.68%)	129 (70.11%)	53 (65.43%)		
Unknown	68 (25.66%)	42 (22.83%)	26 (32.10%)		
Residential address (Shenzhen)	182	129	53	5.7754	0.6724
Baoan	29 (15.93%)	19 (14.73%)	10 (18.87%)		
Futian	22 (12.09%)	14 (10.85%)	8 (15.09%)		
Guangming	11 (6.04%)	8 (6.20%)	3 (5.66%)		
Longgang	50 (27.47%)	38 (29.46%)	12 (22.64%)		
Longhua	15 (8.24%)	12 (9.30%)	3 (5.66%)		
Luohu	30 (16.48%)	21 (16.28%)	9 (16.98%)		
Nanshan	17 (9.34%)	12 (9.30%)	5 (9.43%)		
Yantian	5 (2.75%)	2 (1.55%)	3 (5.66%)		
Others	3 (1.65%)	3 (2.33%)	0 (0.00%)		

Bold font: P value is close to significant. ^†^P value is significant. *Worse represents recurrent, aggravated, or dead. ^#^Under treatment represents first treatment and follow-up treatment.

### Phenotypic drug susceptibility testing

Out of 282 MTB samples, 244 had pDST for four first-line drugs [INH, RFP, rifabutin (RFB), and ethambutol (EMB)] and eight second-line drugs [LFX, MFX, cycloserine (Cs), amikacin (AK), kanamycin (Km), streptomycin (Sm), ethionamide (Eto), and para-aminosalicylic acid (PAS)] (**Supplementary Table 5**). The phenotypic drug-resistant rates on a sample level were 29.10% for INH, 18.85% for RFP, 13.52% for RFB, 16.80% for EMB, 16.80% for LFX, 17.62% for MFX, 11.48% for Cs, 4.10% for AK, 6.15% for Km, 11.48% for Sm, 4.10% for Eto, and 2.46% for PAS. For patients (excluding patients with inconsistent drug resistance in different samples), the overall phenotypic drug-resistant rate with at least one drug resistance was 38.46% (50/130), including 21 DR (16.15%, 21/130), 5 MDR (excluded Pre-XDR) (3.85%, 5/130), and 24 Pre-XDR (18.46%, 24/130) (**Supplementary Table 6**). The overall MDR rate (MDR + Pre-XDR) in patients was 22.31% (29/130) (12.00% (9/75) for new patients and 40.82% (20/49) for retreatment patients).

### Molecular drug susceptibility testing

Genotype-based mDST was obtained by comparing the SNPs in each sample with known drug-resistant mutations reported in the scientific literatures. Totally 16 drugs [five first-line drugs (INH, RFP, RFB, EMB, pyrazinamide (PZA)] and 11 second-line drugs [LFX, MFX, linezolid, clofazimine (Cfz), AK, capreomycin (Cm), Km, Sm, Eto, PAS, and protionamide (PTO)] were included in our study. The mDST in samples showed that the molecular-drug resistant rates were 37.59% (106/282) for INH; 34.40% (97/282) for RFP; 25.18% (71/282) for RFB; 28.01% (79/282) for EMB; 21.28% (60/282) for PZA; 24.11% (68/282) for LFX and MFX; 1.77% (5/282) for linezolid; 1.06% (3/282) for Cfz; 4.61% (13/282) for AK, Cm and Km; 24.47% (69/282) for Sm; 5.67% (16/282) for Eto; 1.77% (5/282) for PAS; and 6.74% (19/282) for PTO. After eliminating patients with inconsistent drug resistance in different samples, the overall molecular drug-resistant rate with at least one drug resistance in patients was 49.43% (86/174), which was higher than that in pDST. We identified 22 DR (12.64%, 22/174), 4 RR (excluded MDR, Pre-XDR, and XDR) (2.30%, 4/174), 17 MDR (excluded Pre-XDR and XDR) (9.77%, 17/174), 40 Pre-XDR (23.00%, 40/174), and 3 XDR (1.72%, 3/174) (**Supplementary Table 6**). Excepting DS and DR, the percentages of RR, MDR, Pre-XDR, and XDR were higher than those in pDST. The overall MDR rates (MDR + Pre-XDR + XDR) were 34.48% (60/174) (20.41% (20/98) for new patients and 58.21% (39/67) for retreatment patients), which was 1.55 times than that of pDST. Comparing mDST with pDST for 11 shared drugs on a sample level, they are highly consistent (93.00% overall accuracy) (**Table 2**).

**Table 2:** The comparison of genotype-based DST with culture-based DST, which was used as the gold standard.

Drug	Total	TP	FN	FP	TN	FPR	FNR	Sensitivity	Specificity	Accuracy	PPV	NPV
AK	244	9	1	2	232	0.85%	10.00%	90.00%	99.15%	98.77%	81.82%	99.57%
EMB	244	38	3	19	184	9.36%	7.32%	92.68%	90.64%	90.98%	66.67%	98.40%
Eto	244	5	5	8	226	3.42%	50.00%	50.00%	96.58%	94.67%	38.46%	97.84%
LFX	244	39	2	7	196	3.45%	4.88%	95.12%	96.55%	96.31%	84.78%	98.99%
INH	244	66	5	15	158	8.67%	7.04%	92.96%	91.33%	91.80%	81.48%	96.93%
Km	244	11	4	0	229	0.00%	26.67%	73.33%	100.00%	98.36%	100.00%	98.28%
MFX	244	38	5	8	193	3.98%	11.63%	88.37%	96.02%	94.67%	82.61%	97.47%
PAS	244	2	4	1	237	0.42%	66.67%	33.33%	99.58%	97.95%	66.67%	98.34%
RFB	244	23	10	27	184	12.80%	30.30%	69.70%	87.20%	84.84%	46.00%	94.85%
RFP	244	43	3	28	170	14.14%	6.52%	93.48%	85.86%	87.30%	60.56%	98.27%
Sm	244	27	1	30	186	13.89%	3.57%	96.43%	86.11%	87.30%	47.37%	99.47%
Total	2684	301	43	145	2195	6.20%	12.50%	87.50%	93.80%	**93.00%**	67.49%	98.08%

TP, true positive; FN, false negative; FP, false positive; TN, true negative; FPR, false positive rate; FNR, false negative rate; PPV, positive predictive value; NPV, negative predictive value.

### Drug-resistant mutations

Comparing mDST to pDST, we identified false negative and false positive samples and inspected resistant mutations in these samples (**Supplementary Table 7**).

#### First-line drugs

To INH, five of 71 phenotypically resistant samples did not have known resistant mutations and 15 of 173 phenotypically susceptible samples had known resistant mutations (12 *katG*:S315T and 3 *fabG1*:-15C/T). The two mutations above also were reported as resistant mutations in catalogue of mutations in *M. tuberculosis* complex (WHO, 2021b). To RFB, 10 of 33 phenotypically resistant samples did not have known resistant mutations whereas 27 of 211 phenotypically susceptible samples had known resistant mutations (23 *rpoB*:S450L, 2 *rpoB*:H445Y, 1 *rpoB*:H445D, and 1 *rpoB*:H445R). For RFP, three of 46 phenotypically resistant samples did not have known resistant mutations whereas 28 of 198 phenotypically susceptible samples had known resistant mutations (20 *rpoB*:S450L, 3 *rpoB*:L452P, 1 *rpoB*:D435V, 1 *rpoB*:H445D, 1 *rpoB*:H445R, 1 *rpoB*:D545E, and 1 *rpoB*:D435G). For EMB, three of 41 phenotypically resistant samples did not have any known resistant mutation and 19 of 203 phenotypically susceptible samples had known resistant mutations (4 *embB*:M306V, 4 *embB*:M306I, 3 *embB*:M306L, 3 *embB*:G406D, 2 *embA*:-12C/T, 1 *embB*:D354A, 1 *embB*:Q497P, and 1 *embB*:H1002R). The first five of seven mutations above in 26 of 28 RFP false positive samples and the first seven of eight mutations above in 18 of 19 EMB false positive samples also were resistant mutations in catalogue of mutations in the *M. tuberculosis* complex (WHO, 2021b).

#### Second-line drugs

Two of 41 phenotypically resistant samples to LFX did not have known resistant mutations, and seven of 203 phenotypically susceptible samples to LFX had known resistant mutations (two *gyrA*:D94A, two *gyrA*:D94N, two *gyrA*:A90V, and one *gyrA*:D94G), whereas five of 43 phenotypically resistance samples to MFX did not have known resistant mutations and eight of 201 phenotypically susceptible samples to MFX had known resistant mutations (three *gyrA*:D94N, two *gyrA*:D94A, two *gyrA*:A90V, and one *gyrA*:D94G). For AK, one of 10 phenotypically resistant samples did not have any known resistance-related mutation whereas two of 234 susceptible samples had *rrs*:A1401G mutation. For Km, there were four phenotypically resistant samples without any known resistant mutation. For Sm, there was one of 28 phenotypically resistant samples without known resistant mutations, whereas 30 of 216 phenotypically susceptible samples had known resistant mutations (6 *rrs*:514A/C, 17 *rpsL*:K43R, 6 *rpsL*:K88R, and *gidB*:A134E). For Eto, five of 10 phenotypically resistant samples did not identify known resistant mutations and eight of 234 phenotypically susceptible samples identified *fabG1*:-15C/T mutation. For PAS, four of six phenotypically resistant samples had none of known resistant mutations and one of 238 phenotypically susceptible samples had known resistant mutation *thyA*:R235P. All mutations above except for *thyA*:R235P in PAS also were resistant mutations with significant association in catalogue of mutations in the *M. tuberculosis* complex (WHO, 2021b).

### Comparison of new treatment samples with retreatment samples

Of the 282 MTB samples, 168 (59.57%, 168/282) were from new patients, 101 (35.82%, 101/282) were from retreatment patients, and the treatment type of the remaining 13 (4.61%, 13/282) could not be ascertained. The percentage of samples in new and retreatment patients varied among lineages (χ2 = 6.7141, P = 0.0348), drug-resistant types (χ2 = 45.6630, P = 6.69e−10 for pDST and χ2 = 59.7620, P = 3.25e−12 for mDST), and treatment outcomes (χ2 = 23.0120, P = 4.01e−05) (**Supplementary Table 8**). The lineage 2 samples were more prevalent in retreatment patients (70.83% (119/168) in new treatment samples vs. 83.17% (84/101) in retreatment samples) probably due to the raised proportion of drug-resistant samples belonging to lineage 2 (pDST: 27.36% (29/106) in new treatment samples vs. 67.16% (45/67) in retreatment samples, χ2 = 24.9720, P = 5.82e−07; mDST: 39.50% (47/119) in new treatment samples vs. 72.62% (61/84) in retreatment samples, χ2 = 20.3890, P = 6.32e−06). For pDST, the percentage of drug-resistant samples was higher in the retreatment group than the new treatment group [61.25% (49/80) vs. 28.48% (43/151)], mainly in Pre-XDR type [36.25% (29/80) vs. 3.97% (6/151)]. Similarly, for mDST, the percentage of drug-resistant samples was also higher in the retreatment group than in the new treatment group [70.30% (71/101) vs. 37.50% (63/168)], mainly in the Pre-XDR type [42.57% (43/101) vs. 7.74% (13/168)]. For treatment outcomes, the percentage of samples with improved outcome was higher in the new treatment group than the retreatment group [69.48% (107/154) vs. 56.52% (52/92)]. Additionally, the percentage of sensitive samples in both improved and stable outcomes was higher in the new treatment group than the retreatment group (pDST: 85.05% (91/107) of new treatment samples vs. 60.47% (26/43) of retreatment samples (χ2 = 9.4160, P = 0.0022) with an improved outcome and 84.62% (33/39) of new treatment samples vs. 50.00% (7/14) of retreatment samples (χ2 = 4.9293, P = 0.0264) with a stable outcome; mDST: 71.03% (76/107) of new treatment samples vs. 31.82% (14/44) of retreatment samples (χ2 = 18.3130, P = 1.87e−05) with an improved outcome and 76.92% (30/39) of new treatment samples vs. 42.86% (6/14) of retreatment samples (χ2 = 4.0351, P = 0.0446) with a stable outcome). Furthermore, there were significant differences in INH-resistant and RFB-resistant mutations between the new treatment group and the retreatment group (χ2 = 8.2277, P = 0.04153 for INH-resistant mutations and χ2 = 8.0630, P = 0.0447 for RFB-resistant mutations). The most frequent mutation in INH and RFB was significantly enriched in the new treatment group than the retreatment group (80.43% vs. 55.26% for *katG*:S315T of INH and 88.89% vs. 76.19% for *rpoB*:S450L of RFB), which indicates the shift of drug-resistant mutations between new treatment and retreatment.

### Transmission network based on WGS

**Figure 2:**
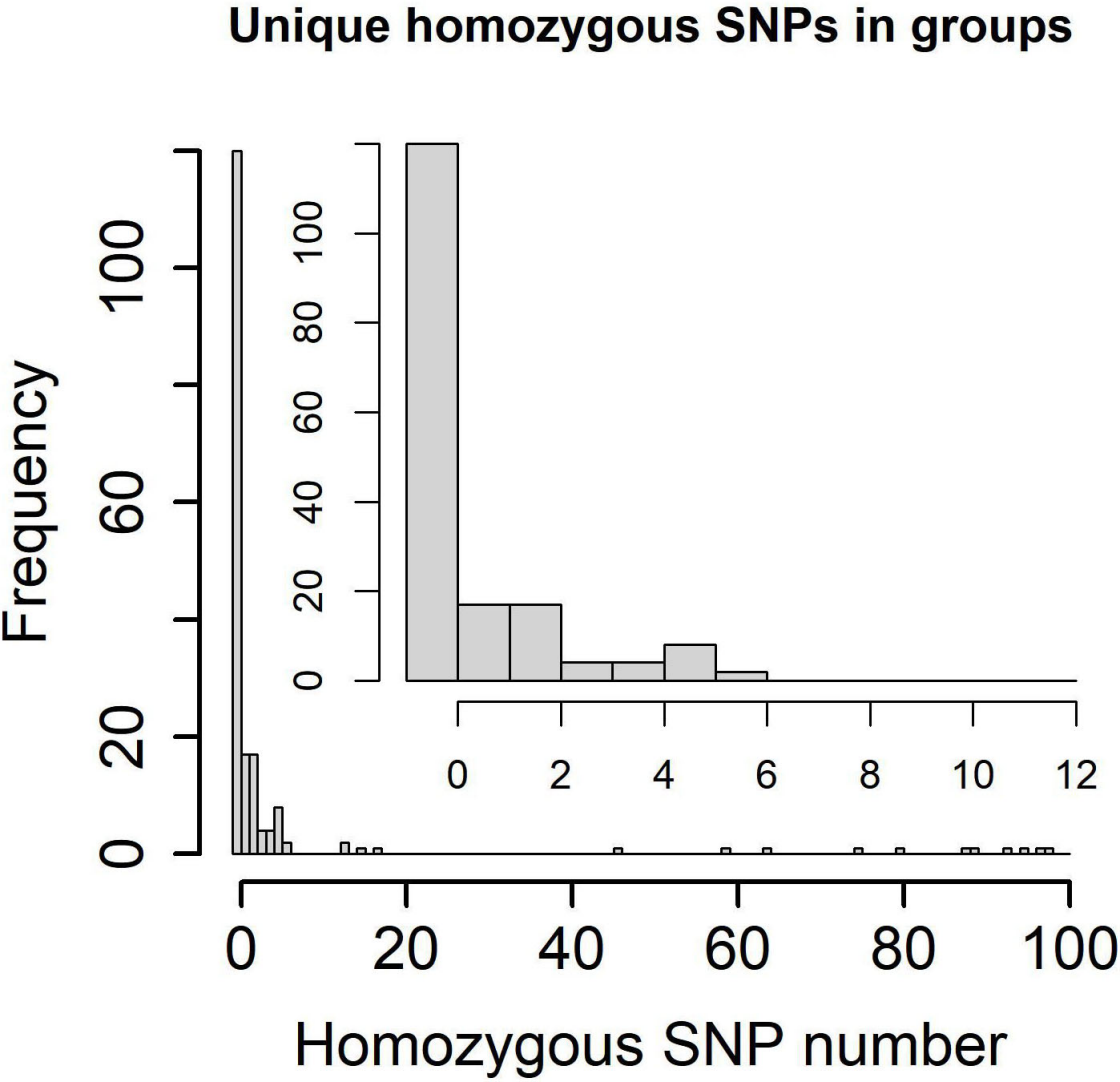
Specific homozygous SNP numbers in genetic clusters and transmission clusters (0~100 SNPs in lower left and 0~12 SNPs in upper right).

The transmission clusters were identified based on the specific SNPs; no more than 12% and 70.21% (198/282) samples (57.46% (104/181) patients) were grouped into 92 transmission clusters with a sample number ranging from two (81 clusters) to five (one cluster) and a patient number ranging from one (87 clusters) to two (five clusters). The number of specific SNPs in these transmission clusters was from 0 to 6 (**Figure 2**). Compared with non-clustered patients (77 patients), clustered patients (104 patients) had more samples (χ2 = 100.3600, P = 1.61e−22), better treatment outcomes (χ2 = 15.3330, P = 0.0041), and more local residential addresses (χ2 = 7.6365, P = 0.0220) (**Table 3**), which implied the recent transmissions. At a sample level, there was no significant difference between non-clustered and clustered samples in lineage, sample type, treatment type, and drug resistance (**Supplementary Table 9**). For clusters with pDST, 2.00% (54 of 75) and 62.67% (47 of 75) clusters were consistent in drug-resistant types and drug-resistant numbers, respectively. For mDST, 94.57% (87 of 92) and 91.30% (84 of 92) clusters were consistent in drug-resistant types and drug-resistant mutations, respectively (**Supplementary Table 10**). The high proportion of identical drug resistance in clusters indicated the recent transmission of drug resistance.

**Table 3:** The clinical characteristics of sequenced TB patients infected with MTB.

Data	Number			X-squared	P value
	Total	Non-clustered	Clustered		
Gender	181	77	104	3.0914	0.0787
Male	131 (72.38%)	50 (64.94%)	81 (77.88%)		
Female	50 (27.62%)	27 (35.06%)	23 (22.12%)		
**Patient samples**	181	77	104	**100.3600**	**1.61e-22**†
1 sample	91 (50.28%)	72 (93.51%)	19 (18.27%)		
2 samples	78 (43.09%)	5 (6.49%)	73 (70.19%)		
3 samples	12 (6.63%)	0 (0.00%)	12 (11.54%)		
Age	181	77	104	5.6790	0.3387
≤20	9 (4.97%)	3 (3.90%)	6 (5.77%)		
21-30	48 (26.52%)	15 (19.48%)	33 (31.73%)		
31-40	34 (18.78%)	17 (22.08%)	17 (16.35%)		
41-50	33 (18.23%)	18 (23.38%)	15 (14.42%)		
51-60	23 (12.71%)	9 (11.69%)	14 (13.46%)		
>60	34 (18.78%)	15 (19.48%)	19 (18.27%)		
Treatment type	181	77	104	2.4529	0.2933
New	101 (55.80%)	38 (49.35%)	63 (60.58%)		
Retreatment	71 (39.23%)	34 (44.16%)	37 (35.58%)		
Unknown	9 (4.97%)	5 (6.49%)	4 (3.85%)		
**Treatment outcome**	181	77	104	**15.333**	**0.0041**†
Improved	96 (53.04%)	29 (37.66%)	67 (64.42%)		
Stable	37 (20.44%)	18 (23.38%)	19 (18.27%)		
Worse*	6 (3.31%)	3 (3.90%)	3 (2.88%)		
Under treatment^#^	41 (22.65%)	26 (33.77%)	15 (14.42%)		
Unknown	1 (0.55%)	1 (1.30%)	0 (0.00%)		
**Residential address**	181	77	104	**7.6365**	**0.0220**†
Non-Shenzhen	13 (7.18%)	3 (3.90%)	10 (9.62%)		
Shenzhen	128 (70.72%)	50 (64.94%)	78 (75.00%)		
Unknown	40 (22.10%)	24 (31.17%)	16 (15.38%)		
Residential address (Shenzhen)	128	50	78	5.8439	0.6647
Baoan	19 (14.84%)	9 (18.00%)	10 (12.82%)		
Futian	14 (10.94%)	6 (12.00%)	8 (10.26%)		
Guangming	8 (6.25%)	2 (4.00%)	6 (7.69%)		
Longgang	38 (29.69%)	15 (30.00%)	23 (29.49%)		
Longhua	12 (9.38%)	7 (14.00%)	5 (6.41%)		
Luohu	21 (16.41%)	7 (14.00%)	14 (17.95%)		
Nanshan	12 (9.38%)	3 (6.00%)	9 (11.54%)		
Yantian	2 (1.56%)	0 (0.00%)	2 (2.56%)		
Others	2 (1.56%)	1 (2.00%)	1 (1.28%)		
Drug resistance of pDST^$^	90	5	85	3.6	0.3080
DR^&^	15 (16.67%)	1 (20.00%)	14 (16.47%)		
DS	40 (44.44%)	4 (80.00%)	36 (42.35%)		
Inconsistency	19 (21.11%)	0 (0.00%)	19 (22.35%)		
Others	16 (17.78%)	0 (0.00%)	16 (18.82%)		
Drug resistance of mDST^$^	90	5	85	3.6519	0.1611
DR^&^	34 (37.78%)	0 (0.00%)	34 (40.00%)		
DS	49 (54.44%)	4 (80.00%)	45 (52.94%)		
Inconsistency	7 (7.78%)	1 (20.00%)	6 (7.06%)		

^†^P value is significant. *Worse represents recurrent, aggravated, and dead. ^#^Under treatment represents first treatment and follow-up treatment. ^$^Only for patients with two samples or more. ^&^DR includes patients with drug resistance of any anti-TB drugs.

Bold font: P value is less than 0.05.

**Figure 3:**
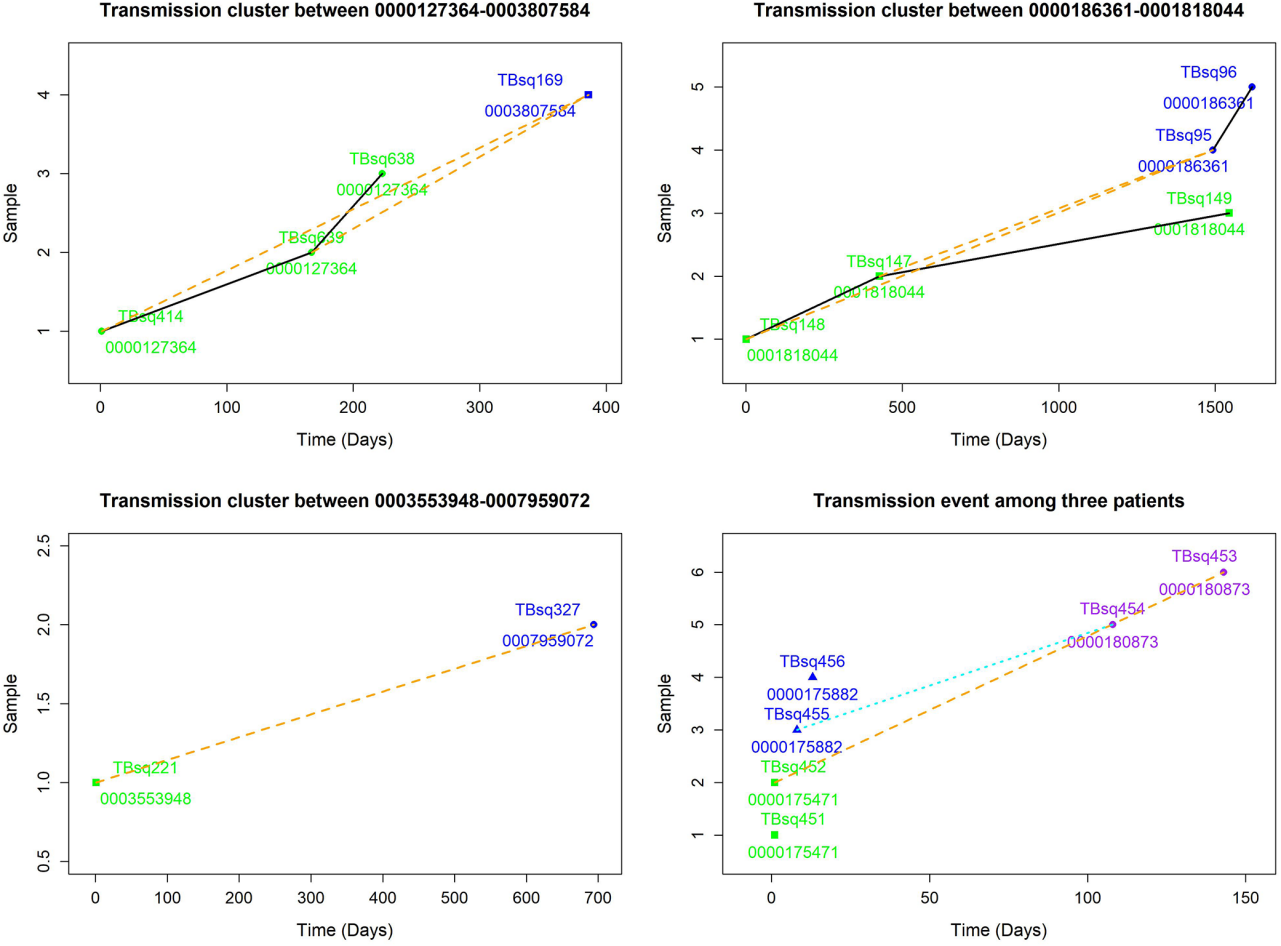
Transmission clusters among multiple patients. The possible transmission relationship was shown. Samples of the same patient were connected with black solid lines, whereas samples between multiple patients were connected with orange dash lines. A cross infection with three patients in two transmission clusters is shown in lower right. Transmission cluster A with TBsq452 of patient 0000175471 and TBsq453 of patient 0000180873. Transmission cluster B with TBsq455 of patient 0000175882 and TBsq454 of patient 0000180873.

Among five transmission clusters with more than one patient, three of them indicated recent transmission of TB between two patients, whereas the other two transmission clusters implied a complicated transmission event (**Figure 3**). The sampling interval between patients in the same transmission cluster was from 100 to 1,492 days (100, 142, 385, 693, and 1,492 days, respectively) with median value 385. The complicated transmission event was related with three patients [patient 0000175471 with TBsq451 (*M. intracellulare*) and TBsq452 samples from pleural fluid and sputum (different species), patient 0000175882 with TBsq455 and TBsq456 samples from pleural fluid and sputum (202 specific SNPs), and patient 0000180873 with TBsq453 and TBsq454 samples from bronchoalveolar lavage fluid and pleural fluid (278 specific SNPs)] in two transmission clusters [transmission cluster A with TBsq452 and TBsq453 (0 specific SNP) and transmission cluster B with TBsq455 and TBsq454 (1 specific SNP)]. Two samples of later infected patient 0000180873 were clustered separately with one sample of previously infected patients, which indicated that later infected patients possibly infected with TB from previous infected patients or all of them were transmitted from the same exposures. The transmission network indicated that uncontrolled transmission existed in the community, even cross transmission.

### Drug-resistant transmission

**Figure 4:**
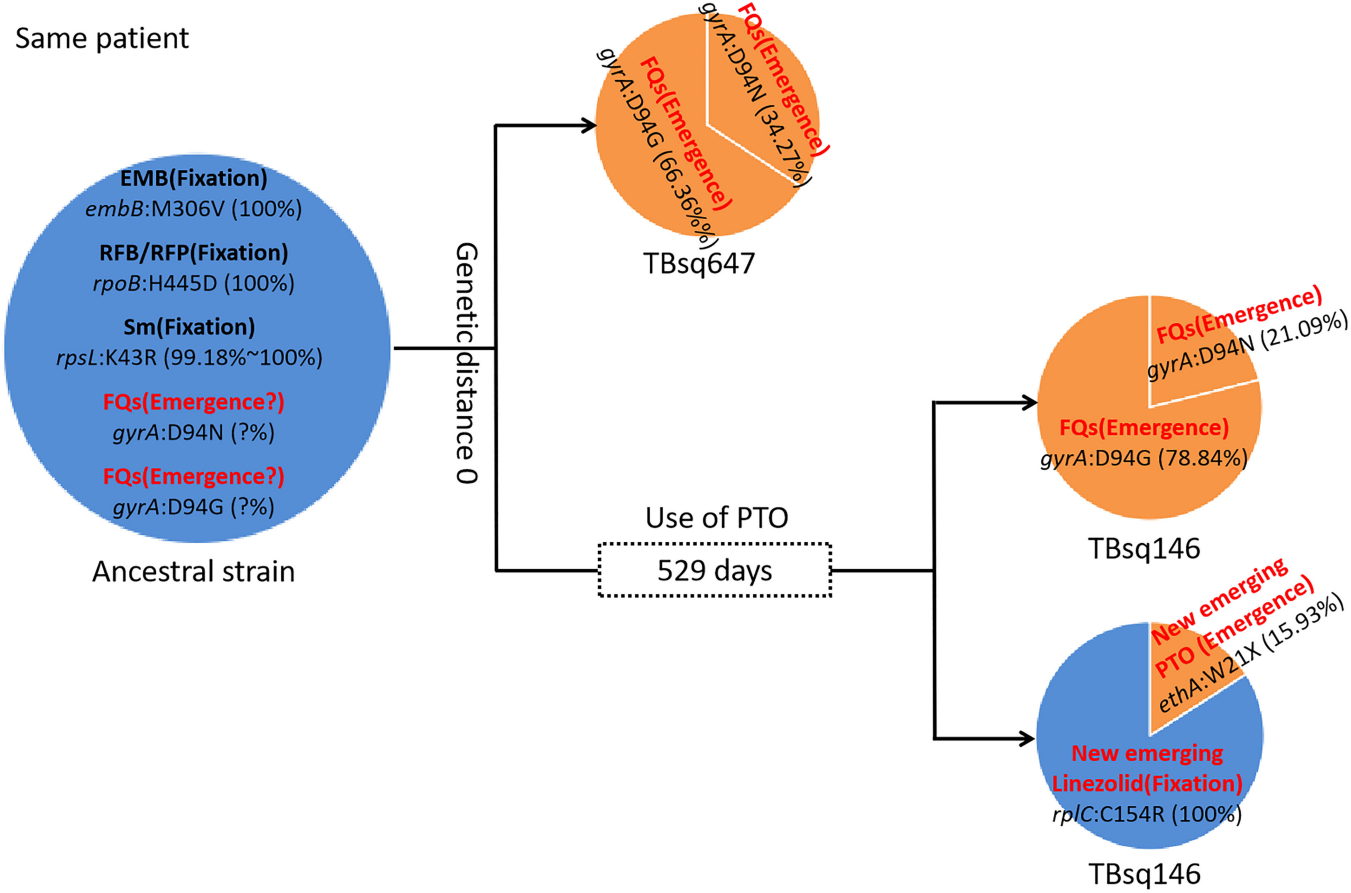
Drug-resistant mutations between TBsq146 and TBsq647 samples of the same patient in the same cluster. The ancestral strain had fixed drug resistance of EMB, RFB/RFP, and Sm and possible emerged drug resistance of FQs. In earlier sample TBsq647, *gyrA*:D94N and *gyrA*:D94G mutations ensured 100% drug resistance for FQs. In later sample TBsq146 after 529 days, the frequency of two drug-resistant mutations for FQs changed, but 100% drug resistance was still maintained. Furthermore, new emerged drug resistance for PTO and linezolid in TBsq146 was found.

**Figure 5:**
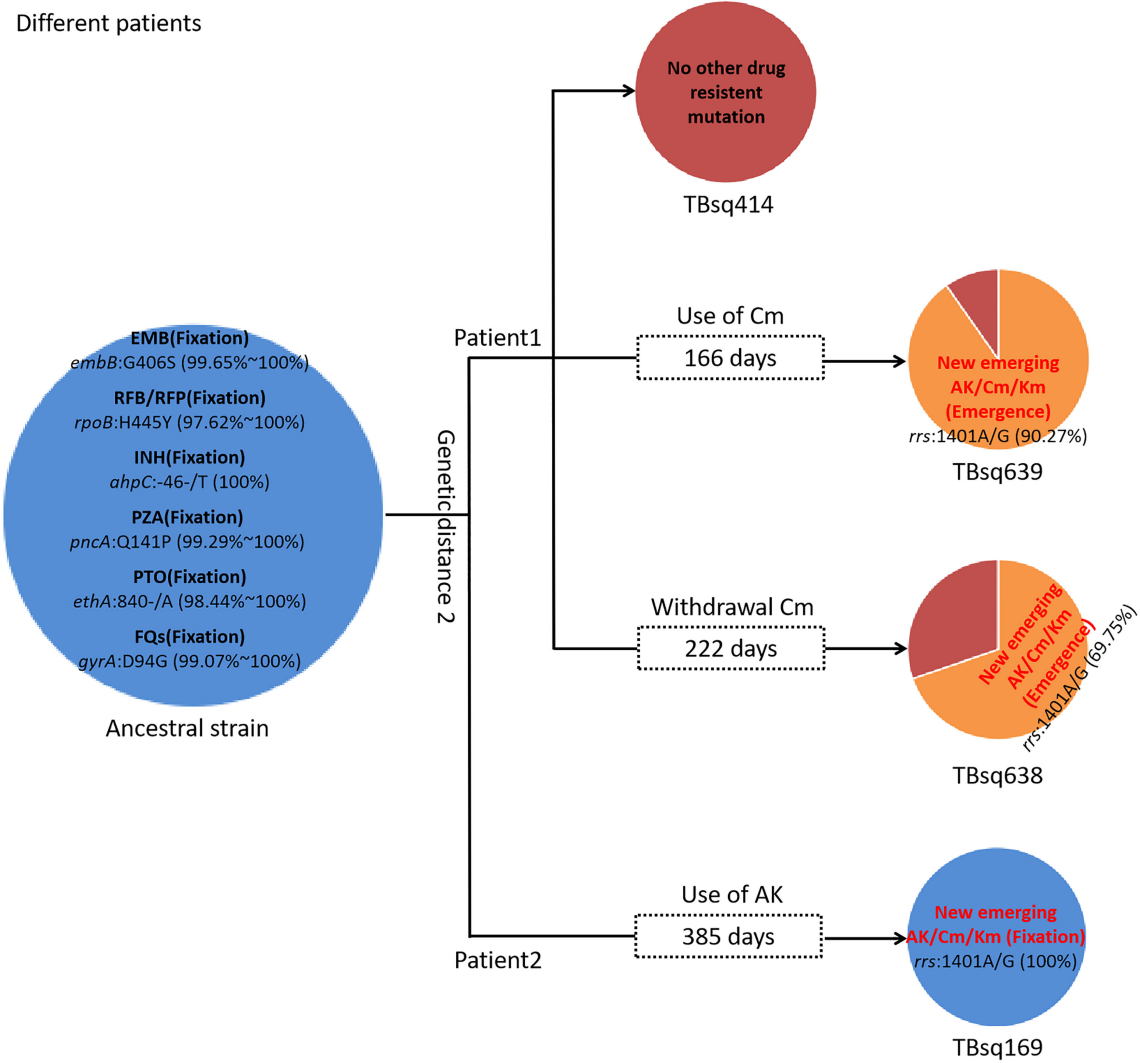
Drug-resistant mutations among TBsq414, TBsq639, and TBsq638 samples of patient 1 and TBsq169 sample of patient 2 in the same cluster. The ancestral strain had fixed drug resistance of EMB, RFB/RFP, INH, PZA, PTO, and FQs. Drug resistance of AK/Cm/Km changed along with use of Cm in patient 1 and AK in patient 2.

To assess recent transmission of drug resistance, drug-resistant mutations of each sample were extracted and 45 of 92 transmission clusters (48.91%) [98 of the 198 samples (49.49%)] had drug-resistant mutations. There were 36 of 45 drug-resistant transmission clusters (80.00%) that were identical for drug-resistant mutations in their corresponding samples, which confirmed possible recent transmission of drug-resistant strains (**Supplementary Table 11**). Two of 36 transmission clusters contained concordant resistant mutations with different allele frequencies in different samples (INH:*ahpC*:-52C/T with 100.00% and 94.42% mutant allele frequencies for samples TBsq268 and TBsq664 in one cluster and FQs:*gyrA*:D94Y with 99.45% and 92.21% mutant allele frequencies for samples TBsq315 and TBsq666 in the other cluster), which suggested that these mutations emerged recently and were selected for gaining resistance. The other nine drug-resistant transmission clusters had discordant resistant mutations among samples (**Supplementary Table 12**). It indicated that some TB strains initiated new drug-resistant mutations recently. For example, in the cluster containing TBsq146 and TBsq647 samples of patient 0005774802, only TBsq146 had *rplC*:C154R mutation with 100.00% mutant allele frequency for linezolid resistance and *ethA*:W21X mutation with 15.93% mutant allele frequency for PTO resistance along with taking PTO. In addition, the concordant mutations *gyrA*:D94N and *gyrA*:D94G for FQ resistance had different mutant allele frequencies in different samples of this cluster. The mutant allele frequencies of mutation *gyrA*:D94N and *gyrA*:D94G were 21.09% and 78.84% for sample TBsq146, and 34.27% and 66.36% for sample TBsq647. This observation indicated that both mutations appeared recently in different subclones and the two mutations were complementary for FQ resistance of both samples (**Figure 4**). Another example was the cluster with the TBsq169 sample of patient 0003807584 and TBsq414, TBsq638, and TBsq639 samples of patient 0000127364. Three samples in this cluster developed resistance for AK, Km and Cm by *rrs*:1401A/G mutation, and mutant allele frequencies of *rrs*:1401A/G were 90.27%, 69.75%, and 100.00% for TBsq639, TBsq638, and TBsq169, respectively, which were consistent with use of Cm or AK (**Figure 5**).

### Comparison of simultaneous sampling patients with continuous sampling patients

According to the sampling interval of patients, we divided patients with multiple samples into SS patients [114 samples from 55 patients, the median of sampling interval time was 3 days (interquartile range, 1–12)] and CS patients [45 samples from 21 patients, the median of sampling interval time was 222 days (interquartile range, 126–846)]. Comparing SS to CS group, the significant differences were found in treatment types (χ2 = 34.2790, *P* = 3.60e−08), treatment outcomes (χ2 = 19.8450, *P* = 0.0005), residential addresses (χ2 = 13.0090, P = 0.0015), and drug-resistant types (χ2 = 9.6863, *P* = 0.0214 for pDST and χ2 = 6.7324, *P* = 0.0345 for mDST) (**Table 4**). The SS patients were more prevalent in new treatment type (78.18% (43/55) in the SS group vs. 14.29% (3/21) in the CS group), improved or stable outcomes (70.91% (39/55) improved and 21.82% (12/55) stable patients in the SS group vs. 47.62% (10/21) improved and 9.52% (2/21) stable patients in the CS group), and drug-susceptible type (pDST: 52.73% (29/55) in the SS group vs. 14.29% (3/21) in the CS group; mDST: 58.18% (32/55) in the SS group vs. 33.33% (7/21) in the CS group).

**Table 4:** The clinical characteristics of sequenced TB patients belonged to simultaneous sampling (SS) and continuous sampling (CS) categories.

Data	Number		X-squared	P value
	SS	CS		
Gender	55	21	0.0817	0.7750
Male	41 (74.55%)	17 (80.95%)		
Female	14 (25.45%)	4 (19.05%)		
Patient samples	55	21	0.2519	0.6157
2 samples	51 (92.73%)	18 (85.71%)		
3 samples	4 (7.27%)	3 (14.29%)		
Age	55	21	9.5147	0.0902
≤20	4 (7.27%)	2 (9.52%)		
21-30	22 (40.00%)	3 (14.29%)		
31-40	11 (20.00%)	3 (14.29%)		
41-50	9 (16.36%)	3 (14.29%)		
51-60	6 (10.91%)	6 (28.57%)		
>60	3 (5.45%)	4 (19.05%)		
Treatment type	55	21	**34.2790**	**3.60e-08**
New	43 (78.18%)	3 (14.29%)		
Retreatment	8 (14.55%)	18 (85.71%)		
Unknown	4 (7.27%)	0 (0.00%)		
Treatment outcome	55	21	**19.8450**	**0.0005**
Improved	39 (70.91%)	10 (47.62%)		
Stable	12 (21.82%)	2 (9.52%)		
Worse*	2 (3.64%)	1 (4.76%)		
Under treatment^#^	1 (1.82%)	8 (38.10%)		
Unknown	1 (1.82%)	0 (0.00%)		
Residential address	55	21	**13.0090**	**0.0015**
Non-Shenzhen	6 (10.91%)	1 (4.76%)		
Shenzhen	47 (85.45%)	13 (61.90%)		
Unknown	2 (3.64%)	7 (33.33%)		
Residential address (Shenzhen)	47	13	10.409	0.2375
Baoan	6 (3.64%)	3 (23.08%)		
Futian	5 (3.64%)	1 (7.69%)		
Guangming	4 (3.64%)	1 (7.69%)		
Longgang	19 (3.64%)	1 (7.69%)		
Longhua	3 (3.64%)	0 (0.00%)		
Luohu	4 (3.64%)	2 (15.38%)		
Nanshan	4 (3.64%)	4 (30.77%)		
Yantian	1 (3.64%)	1 (7.69%)		
Others	1 (3.64%)	0 (0.00%)		
Drug resistance of pDST^$^	55	21	**9.6863**	**0.0214**
DR^&^	7 (12.73%)	6 (28.57%)		
DS	29 (52.73%)	3 (14.29%)		
Inconsistency	11 (20.00%)	6 (28.57%)		
Others	8 (14.55%)	6 (28.57%)		
Drug resistance of mDST^$^	55	21	**6.7324**	**0.0345**
DR^&^	21 (38.18%)	10 (47.62%)		
DS	32 (58.18%)	7 (33.33%)		
Inconsistency	2 (3.64%)	4 (19.05%)		
Cluster	55	21	9.07e−31	1.0000
Non-clustered	4 (7.27%)	2 (9.52%)		
Clustered	51 (92.73%)	19 (90.48%)		

*Worse represents recurrent, aggravated, and died. ^#^Under treatment represents first treatment and follow-up treatment. ^$^Only for patients with two samples or more. ^&^DR includes XDR, Pre-XDR, MDR, DR, and RR. SS, simultaneous sampling; CS, continuous sampling.

Bold font: P value is less than 0.05.

Compared with mDST, pDST was more discordant in drug susceptibility of patients [23.40% (11/47) for pDST vs. 3.64% (2/55) for mDST in the SS group (χ2 = 7.2164, *P* = 0.0072) and 40.00% (6/15) for pDST vs. 19.05% (4/21) for mDST in the CS category (χ2 = 1.0127, *P* = 0.3142)]. We inspected all 17 discordant patients in pDST and found that 16 of 17 were concordant in mDST. According to mDST, the two discordant drug resistance patients in the SS category were both infected by MTB and NTM simultaneously, whereas the four discordant drug resistance patients in the CS category developed new drug resistance as time went by. Further analysis identified discordant strains among SS (5/55, 9.09%) and CS (2/21, 9.52%) patients based on genetic clustering, suggesting the presence of the polyclonal infections and reinfections.

### Mixed infection

**Figure 6:**
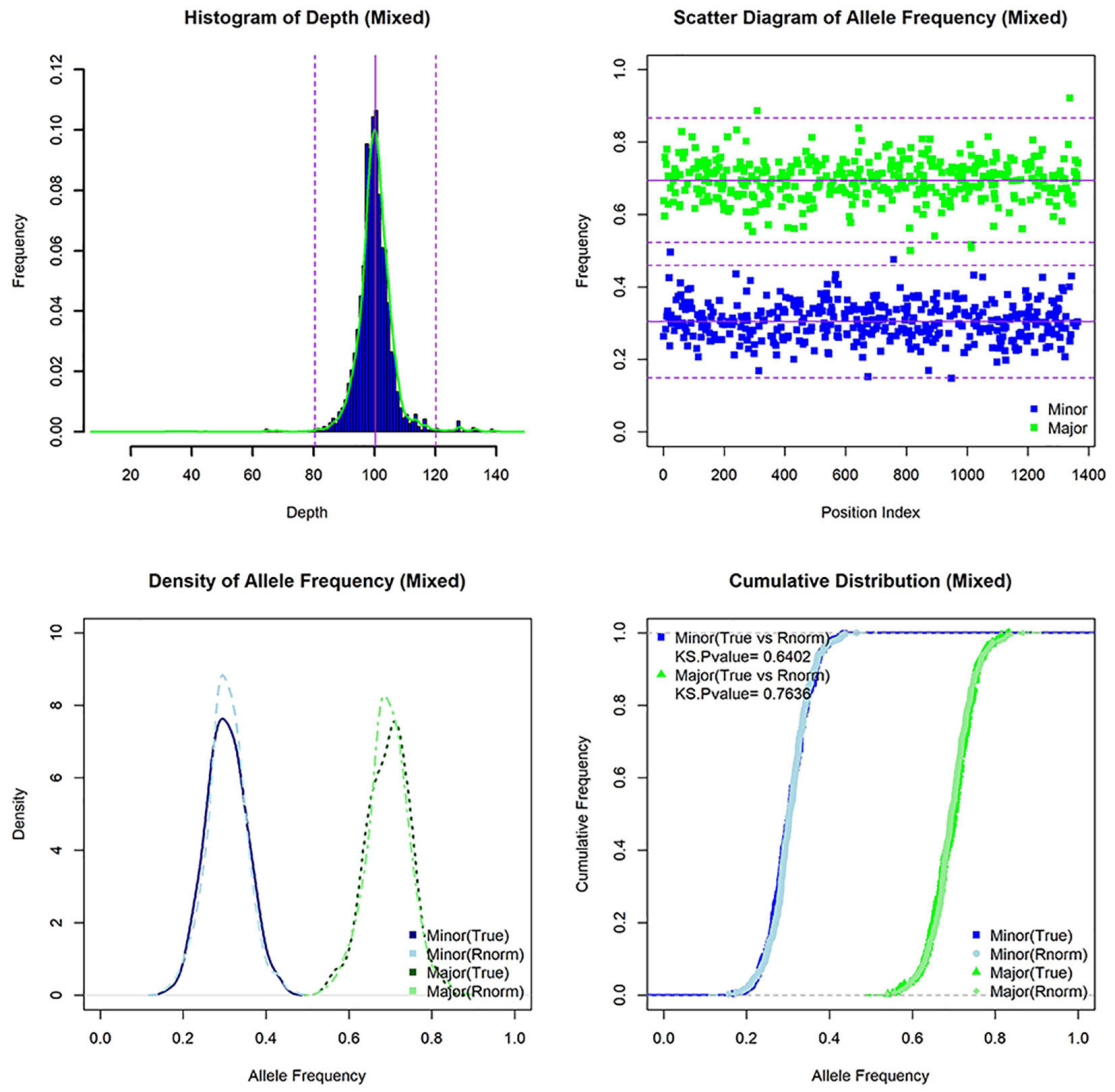
The characteristics of simulated mixed infection sample [lineage 1(30X)+lineage 3(70X)]. Histogram of depth, scatter diagram of allele frequency, density of allele frequency, and cumulative distribution of allele frequency for mixed sample (and random normal distribution data).

**Figure 7:**
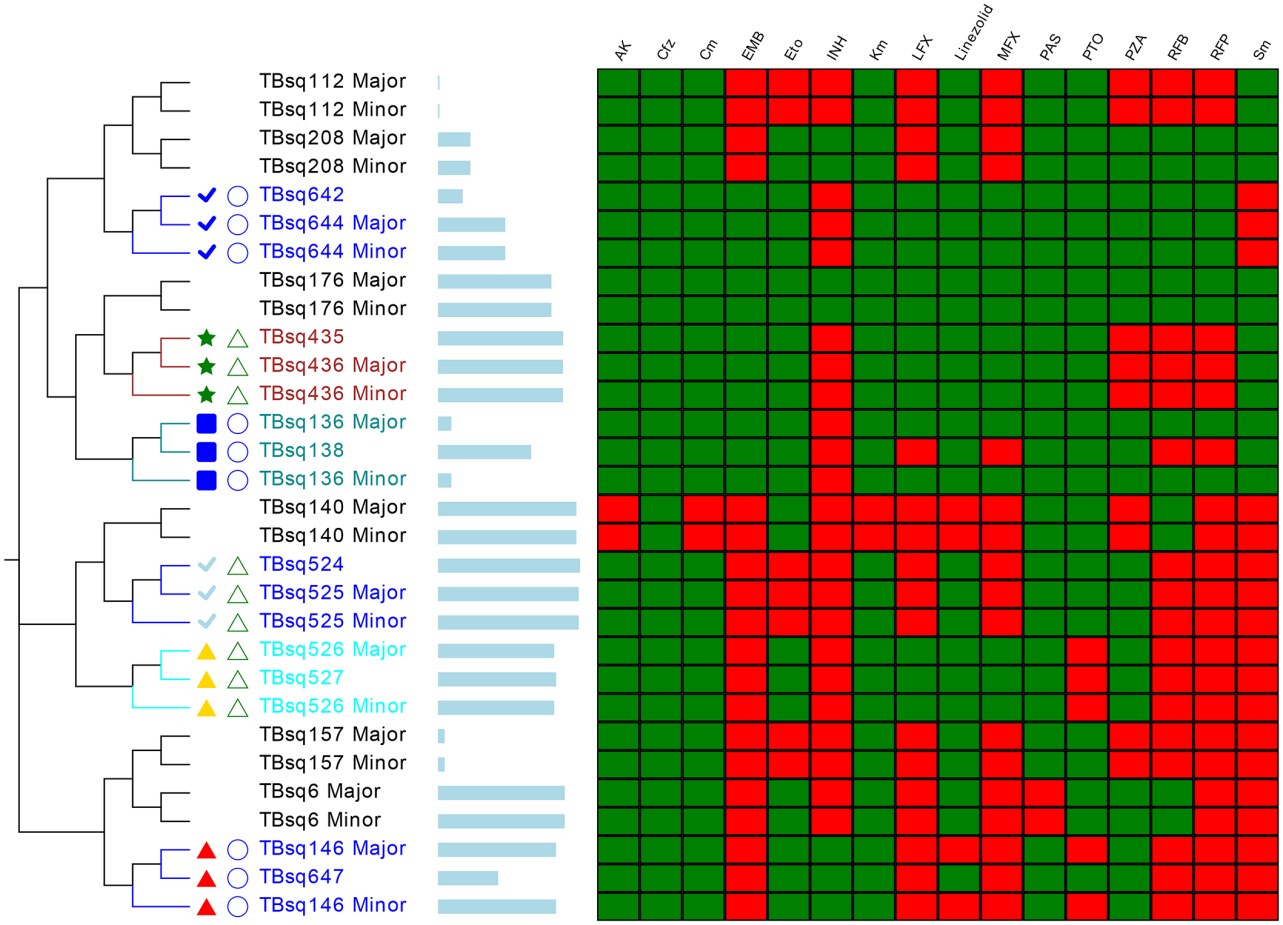
The phylogenetic tree of major, minor, and corresponding pure strains. The different colors on branches and leaf labels indicate different specific SNP numbers in transmission clusters (blue: 0; brown: 1; cyan: 2; dark cyan: 3). Two columns of leaf decorations are shown for patients with more than one samples (unique solid color shape for each patient) and patients in SS (open green triangle) and CS (open blue circle) categories. Relative time of culture-positive sample is shown next with a leaf label with a bar plot (from 1 day to 1,297 days). Genotype-based mDST results are shown with a heatmap (green for susceptible and red for resistance).

To study mixed infection of clinical samples, we established a method for identification of mixed infection based on the hypothesis that allele frequency profiles of heterozygous SNPs in mixed infection and pure samples are normal and non-normal distributions, respectively (**Figure 6**). Using simulated data and artificial mixed clinical data, the accuracy of this method is between 91.36% and 94.59% (**Table 5**). This method relies on the relative ratio of mixed strains, number of heterozygous SNPs, and total SNPs. Based on this method, 12 of 282 (4.26%) clinical samples (12 of 181 patients) were identified as mixed infection samples (**Supplementary Table 13**). Among them, six patients had both mixed infection and pure infection samples together. Phylogeny analysis revealed that major strain of mixed samples was closer to corresponding pure strain than minor strain (**Figure 7**). Furthermore, three of these patients belonging to the SS category had the same drug-resistant mutations whereas two of three patients belonging to the CS category had different drug-resistant mutations in mixed and pure samples, which reflected the change of drug resistance along with adjustment of drug prescription (drug resistance of PTO in one patient and drug resistance of RFP/RFB and FQs (LFX/MFX) in another patient) (**Supplementary Table 14**).

**Table 5:** Method evaluation for identification of mixed infection.

Datasets	Simulated data	Artificial mixed data	Combined
Total	81 (6 non-mix+75 mix)	148 (8 non-mix+140 mix)	229 (14 non-mix + 215 mix)
True positive	68	132	200
False negative	7	8	15
False positive	0	0	0
True negative	6	8	14
False positive rate	0.00%	0.00%	0.00%
False negative rate	9.33%	5.71%	6.98%
Sensitivity	90.67%	94.29%	93.02%
Specificity	100.00%	100.00%	100.00%
Accuracy	91.36%	94.59%	93.45%

## Discussion

To study the transmission pattern and drug resistance of MTB in the only municipal hospital for infectious diseases in Shenzhen, TB patients diagnosed during 2015–2018 were enrolled for WGS. These patients mainly were refractory patients referred from distinct infectious disease hospitals. Sequence data were obtained for 184 of 265 patients (286 of 500 samples). The sequenced patients had more samples (83.70% (154/184) in sequenced patients vs. 69.14% (56/81) in non-sequenced patients) and better treatment outcomes (55.43% (102/184) in sequenced patients vs. (45.68% (37/81) in non-sequenced patients). Based on species markers in rRNA genes, 282 of 286 (98.60%) sequenced samples were identified as MTB.

Culture-based pDST of 12 drugs was done for 244 of 282 MTB samples. Meanwhile, mDST of 16 drugs was done for 282 MTB samples by comparing SNPs with known drug-resistant mutations. The patients’ overall drug-resistant rates with at least one drug resistance were 38.46% and 49.43%, and the MDR rates were 22.31% and 34.48% for pDST and mDST, respectively. The MDR rates of new and retreatment patients were 12.00% and 40.82% in pDST and 20.41% and 58.21% in mDST, respectively. In Shenzhen, population-based studies have reported varying MDR rates. In Bao’an District, the MDR rates for all, new, and retreatment patients were 6.7%, 3.8%, and 20.4%, respectively, between 2014 and 2017 (Yang et al., 2021). In Longhua District, these rates were 4.4%, 3.7%, and 18.3% between 2018 and 2021 (Mijiti et al., 2023), whereas in the whole city of Shenzhen, these rates were 5.7%, 3.8%, and 27.1% during 2012–2020 (Lecai et al., 2021). Previous studies also estimated that the overall MDR rate was 5.08% in Shenzhen during 2013–2017 (Jiang et al., 2020) and 6.77% in China from January to December 2013 (Liu et al., 2024). Meta-analysis in China revealed that the MDR rates for new and retreatment patients were 5.4% and 28.5% in 2012 (Ding et al., 2017), and 4.8% and 26.3% between 2012 and 2015, respectively (Duan et al., 2016). Globally, the MDR rates for new and retreatment cases were 5.25% and 17.02% in 2019, respectively, according to the global tuberculosis report (WHO, 2020). The high drug-resistant rate observed in our study may be attributed to the inclusion of a broader range of drugs (12 for pDST and 16 for mDST) and the specific sample collection hospital. As Shenzhen’s only municipal infectious disease hospital, the enrolled patients were mainly refractory TB cases referred from district-level infectious disease hospitals, often with higher rates of drug resistance and mixed infections (Qu et al., 2024). Furthermore, 100.00% and 93.75% of RR patients in our study were MDR patients based on pDST and mDST, respectively, exceeding the global rate reported in global TB report 2020 (74% of RR patients with MDR) (WHO, 2020). Comparing mDST with pDST for 11 shared drugs, they are highly consistent (93.00% overall accuracy). Using pDST as the gold standard, the discrepancy was mainly focused on drug resistance than drug sensitivity (12.50% (43/344) in drug resistance vs. 6.20% (145/2340) in drug sensitivity, χ2 = 17.3390, P = 3.13e−05). We also inspected all drug-resistant mutations (RFP, INH, EMB, FQs, AK, Sm, and Eto) and found that most mutations [97.27%, (107/110)] in false positive samples were also reported in the catalogue of mutations in the *M. tuberculosis* complex (WHO, 2021b).

Comparing the new treatment samples, lineage 2 and drug resistance based on mDST were more prevalent in retreatment samples. It seems that non-lineage 2 samples tend to be drug sensitive (62.32% (43/69) in non-lineage 2 samples vs. 48.36% (103/213) in lineage 2 samples, χ2 = 3.5290, *P* = 0.0603). In addition, the improved and stable treatment outcomes were higher in new treatment samples than retreatment samples. Furthermore, we found that the most frequent resistant mutations in INH and RFB were more enriched in new treatment samples than retreatment samples.

Totally 92 transmission clusters with 70.21% samples (57.46% patients) were identified. Among of them, 87 clusters had only one patient and five clusters had more than one patients. It is worth mentioning that two clusters involved into a complicated transmission event related with three patients. The two samples of later infected patients were separately clustered with one sample of two early infected patients, respectively, which indicated a possible cross infection. There were 36 of 45 transmission clusters (80.00%) with drug-resistant mutations that were completely concordant in all samples, which suggested the transmission of drug resistance. Nine of 45 transmission clusters had discordant drug-resistant mutations and implied that different samples produced different mutations recently.

Patients with more than one sample were classified as SS and CS patients according to sampling interval. The treatment types, treatment outcomes, residential addresses, and drug-resistant types varied between SS and CS categories. More interestingly, compared with pDST, mDST was more consistent in both SS and CS categories, which showed greater accuracy in mDST than pDST.

Mixed infection significantly influences epidemiology investigation, evolutionary analysis, and drug resistance. However, the identification of mixed infection remains a major challenge in *M. tuberculosis* study (Meehan et al., 2019). Although some mathematical approaches were developed based on the Bayesian model (Sobkowiak et al., 2018) or phylogenetic method (Gan et al., 2016), no convenient, unbiased tool currently exists for identifying mixed infection. We developed a new method based on characteristics of heterozygous SNPs and two-sample Kolmogorov–Smirnov test. Using simulated data and artificial mixed clinical data, the accuracy of this method is between 91.36% and 94.59%. There were 12 of 282 samples identified as mixed infection samples, and the major strain of mixed sample was closer to the corresponding pure sample in the same patient. Moreover, the difference of drug resistance among samples is mainly due to the long sampling interval of time and change of drug prescription. Despite its strengths, our method has some limitations. First, the detection of mixed infections is limited by the ratio of minority strain (e.g., ≤5%) and the number of heterozygous SNPs (e.g., ≤18). Second, the two-sample Kolmogorov–Smirnov test performs better with samples containing more heterozygous SNPs. Third, the method is restricted to identifying mixed infection samples with two strains.

In summary, using WGS, we conducted a retrospective study of MTB infections in Shenzhen Third People’s Hospital. The drug resistance rates were analyzed and compared with previous studies. We also assessed the clustering characteristics of MTB and revealed MTB transmission pattern. As the patients were mainly refractory patients, this study provides the opportunity to investigate the polyclonal infections and track the change of MTB in long-term treatment.

## Data Availability

Raw data was deposited in the Genome Sequence Archive (GSA: CRA018968) at National Genomics Data Center, Beijing Institute of Genomics, Chinese Academy of Sciences/China National Center for Bioinformation, which can be accessed at https://bigd.big.ac.cn/gsa.
