# Supplementary material for: Whole-genome sequencing reveals transmission pattern and drug resistance of Mycobacterium tuberculosis intra- or inter-hosts
Source: Front Cell Infect Microbiol. 2025 Jan 21;14:1488547. doi: 10.3389/fcimb.2024.1488547 (PMC11790449; doi:10.3389/fcimb.2024.1488547)
Supplement: Supplementary file 7 [file Table6.docx]

Supplementary Table 6. The comparison of new treatment patients to retreatment patients with sequence data.

| Data | Number | | | X-squared | P value |
| --- | --- | --- | --- | --- | --- |
|  | Total§ | New treatment | Retreatment |  |  |
| Lineage | 181 | 101 | 71 | 3.4326 | 0.3296 |
| Lineage1 | 2(1.10%) | 1(0.99%) | 0(0.00%) |  |  |
| Lineage2 | 140(77.35%) | 74(73.27%) | 60(84.51%) |  |  |
| Lineage4 | 36(19.89%) | 24(23.76%) | 10(14.08%) |  |  |
| Inconsistency | 3(1.66%) | 2(1.98%) | 1(1.41%) |  |  |
| Cluster | 181 | 101 | 71 | 1.1126 | 0.2915 |
| Non-clustered | 78(43.09%) | 39(38.61%) | 34(47.89%) |  |  |
| Clustered | 103(56.91%) | 62(61.39%) | 37(52.11%) |  |  |
| Treatment outcome | 180 | 101 | 71 | **17.8780** | **4.66e-4**† |
| Improved | 96(53.33%) | 60(59.41%) | 29(40.85%) |  |  |
| Stable | 37(20.56%) | 26(25.74%) | 11(15.49%) |  |  |
| Worse* | 6(3.33%) | 2(1.98%) | 3(4.23%) |  |  |
| Under treatment^#^ | 41(22.78%) | 13(12.87%) | 28(39.44%) |  |  |
| Culture-based drug resistance^$^ | 149 | 86 | 54 | **25.8120** | **3.45e-05**† |
| Pre-XDR | 24(16.11%) | 5(5.81%) | 19(35.19%) |  |  |
| MDR^⁑^ | 5(3.36%) | 4(4.65%) | 1(1.85%) |  |  |
| DR | 21(14.09%) | 10(11.63%) | 11(20.37%) |  |  |
| DS | 80(53.69%) | 56(65.12%) | 18(33.33%) |  |  |
| Inconsistency | 19(12.75%) | 11(12.79%) | 5(9.26%) |  |  |
| Genotype-based drug resistance | 181 | 101 | 71 | **32.8970** | **1.10e-05**† |
| XDR | 3(1.66%) | 0(0.00%) | 3(4.23%) |  |  |
| Pre-XDR | 40(22.10%) | 11(10.89%) | 29(40.85%) |  |  |
| MDR | 17(9.39%) | 9(8.91%) | 7(9.86%) |  |  |
| RR^‡^ | 4(2.21%) | 4(3.96%) | 0(0.00%) |  |  |
| DR | 22(12.15%) | 14(13.86%) | 8(11.27%) |  |  |
| DS | 88(48.62%) | 60(59.41%) | 20(28.17%) |  |  |
| Inconsistency | 7(3.87%) | 3(2.97%) | 4(5.63%) |  |  |

Note: §: Total includes new treatment, retreatment and unknown; †: P value is significant; *: Worse represents recurrent, aggravated and dead; #: Under treatment represents first treatment and follow-up treatment; $: Only 149 patients with culture-based DST results for all samples show here; ⁑: MDR excluded Pre-XDR and XDR; ‡: RR excluded MDR, Pre-XDR and XDR.
