# Supplementary material for: Whole-genome sequencing reveals transmission pattern and drug resistance of Mycobacterium tuberculosis intra- or inter-hosts
Source: Front Cell Infect Microbiol. 2025 Jan 21;14:1488547. doi: 10.3389/fcimb.2024.1488547 (PMC11790449; doi:10.3389/fcimb.2024.1488547)
Supplement: Supplementary file 9 [file Table8.docx]

Supplementary Table 8. The comparison of new treatment samples to retreatment samples with sequence data.

| Data | Number | | | X-squared | P value |
| --- | --- | --- | --- | --- | --- |
|  | Total§ | New treatment | Retreatment |  |  |
| Lineage | 282 | 168 | 101 | **6.7141** | **0.0348**† |
| Lineage1 | 6(2.13%) | 5(2.98%) | 0(0.00%) |  |  |
| Culture-based Sensitive | 5 | 5 | 0 | - | - |
| Culture-based Resistant | 1 | 0 | 0 |  |  |
| Genotype-based Sensitive | 6 | 5 | 0 | - | - |
| Genotype-based Resistant | 0 | 0 | 0 |  |  |
| Lineage2 | 213(75.53%) | 119(70.83%) | 84(83.17%) |  |  |
| Culture-based Sensitive | 107 | 77 | 22 | **24.9720** | **5.82e-07**† |
| Culture-based Resistant | 76 | 29 | 45 |  |  |
| Genotype-based Sensitive | 103 | 72 | 23 | **20.3890** | **6.32e-06**† |
| Genotype-based Resistant | 110 | 47 | 61 |  |  |
| Lineage4 | 63(22.34%) | 44(26.19%) | 17(16.83%) |  |  |
| Culture-based Sensitive | 37 | 26 | 9 | 1.79e-31 | 1.0000 |
| Culture-based Resistant | 18 | 14 | 4 |  |  |
| Genotype-based Sensitive | 37 | 28 | 7 | 1.6943 | 0.1930 |
| Genotype-based Resistant | 26 | 16 | 10 |  |  |
| Lineage2 |  |  |  | 1.52e-31 | 1.0000 |
| Beijing sublineage | 203(95.31%) | 113(94.96%) | 80(95.24%) |  |  |
| Others | 10(4.69%) | 6(5.04%) | 4(4.76%) |  |  |
| Cluster | 282 | 168 | 101 | 2.0931 | 0.1480 |
| Non-clustered | 84(29.79%) | 43(25.60%) | 35(34.65%) |  |  |
| Clustered | 198(70.21%) | 125(74.40%) | 66(65.35%) |  |  |
| Culture-based drug resistance^$^ | 244 | 151 | 80 | **45.6630** | **6.69e-10**† |
| Pre-XDR | 35(14.34%) | 6(3.97%) | 29(36.25%) | 3.7670 | 0.1521 |
| 2~5 drugs | 11 | 2 | 9 |  |  |
| 6~9 drugs | 20 | 2 | 18 |  |  |
| >=10 drugs | 4 | 2 | 2 |  |  |
| MDR/RR^⁑^ | 11(4.51%) | 8(5.30%) | 3(3.75%) | 2.1771 | 0.3367 |
| 1~2 drugs | 3 | 3 | 0 |  |  |
| 3~4 drugs | 4 | 3 | 1 |  |  |
| >=5 drugs | 4 | 2 | 2 |  |  |
| DR | 49(20.08%) | 29(19.21%) | 17(21.25%) | 3.7473 | 0.1536 |
| 1 drug | 29 | 16 | 10 |  |  |
| 2 drugs | 14 | 11 | 3 |  |  |
| >=3 drugs | 6 | 2 | 4 |  |  |
| DS | 149(61.07%) | 108(71.52%) | 31(38.75%) |  |  |
| Treatment outcome | 246 | 154 | 92 | **23.0120** | **4.01e-05**† |
| Improved | 159(64.63%) | 107(69.48%) | 52(56.52%) | **9.4160** | **0.0022**† |
| Sensitive | 124 | 91 | 26 |  |  |
| Resistant | 34 | 16 | 17 |  |  |
| Stable | 55(22.36%) | 39(25.32%) | 16(27.39%) | **4.9293** | **0.0264**† |
| Sensitive | 42 | 33 | 7 |  |  |
| Resistant | 13 | 6 | 7 |  |  |
| Worse* | 5(2.03%) | 2(1.30%) | 3(3.26%) | 0.0000 | 1.0000 |
| Sensitive | 4 | 1 | 2 |  |  |
| Resistant | 1 | 1 | 0 |  |  |
| Under treatment^#^ | 27(10.98%) | 6(3.90%) | 21(22.83%) | 1.48e-31 | 1.0000 |
| Sensitive | 17 | 4 | 13 |  |  |
| Resistant | 7 | 1 | 6 |  |  |
| Genotype-based drug resistance | 282 | 168 | 101 | **59.7620** | **3.25e-12**† |
| XDR | 5(1.77%) | 0(0.00%) | 5(4.95%) |  |  |
| Pre-XDR | 56(19.86%) | 13(7.74%) | 43(42.57%) | 4.6598 | 0.0973 |
| 2~5 drugs | 12 | 0 | 12 |  |  |
| 6~9 drugs | 33 | 10 | 23 |  |  |
| >=10 drugs | 11 | 3 | 8 |  |  |
| MDR/RR^⁑^ | 36(12.77%) | 23(13.69%) | 11(10.89%) |  |  |
| 1~2 drugs | 5 | 5 | 0 | 4.2750 | 0.1179 |
| 3~4 drugs | 9 | 7 | 2 |  |  |
| >=5 drugs | 22 | 11 | 9 |  |  |
| DR | 39(13.83%) | 27(16.07%) | 12(11.88%) |  |  |
| 1 drug | 16 | 12 | 4 | 0.6139 | 0.7357 |
| 2 drugs | 13 | 8 | 5 |  |  |
| >=3 drugs | 10 | 7 | 3 |  |  |
| DS | 146(51.77%) | 105(62.50%) | 30(29.70%) |  |  |
| Treatment outcome | 246 | 154 | 92 | **23.0120** | **4.01e-05**† |
| Improved | 159(64.63%) | 107(69.48%) | 52(56.52%) | **18.3130** | **1.87e-05**† |
| Sensitive | 96 | 76 | 14 |  |  |
| Resistant | 63 | 31 | 30 |  |  |
| Stable | 55(22.36%) | 39(25.32%) | 16(27.39%) | **4.0351** | **0.0446**† |
| Sensitive | 38 | 30 | 6 |  |  |
| Resistant | 17 | 9 | 8 |  |  |
| Worse* | 5(2.03%) | 2(1.30%) | 3(3.26%) |  |  |
| Sensitive | 3 | 1 | 1 | 0.0000 | 1.0000 |
| Resistant | 2 | 1 | 1 |  |  |
| Under treatment^#^ | 27(10.98%) | 6(3.90%) | 21(22.83%) | 5.27e-31 | \| 1.0000 \| \| --- \| |
| Sensitive | 12 | 3 | 9 |  |  |
| Resistant | 13 | 3 | 10 |  |  |
| Genotype-based resistant mutation |  |  |  |  |  |
| AK/Cm/Km | 13 | 3 | 10 | 4.07e-30 | 1.0000 |
| rrs:1401A/G | 12(92.31%) | 3(100.00%) | 9(90.00%) |  |  |
| Others | 1(7.69%) | 0(0.00%) | 1(10.00%) |  |  |
| EMB | 89 | 32 | 57 | 7.3788 | 0.2872 |
| embB:M306V | 25(28.09%) | 10(31.25%) | 15(26.32%) |  |  |
| embB:M306I | 20(22.47%) | 8(25.00%) | 12(21.05%) |  |  |
| embB:M306L | 7(7.87%) | 5(15.63%) | 2(3.51%) |  |  |
| embB:G406S | 6(6.74%) | 1(3.13%) | 5(8.77%) |  |  |
| embB:G406D | 6(6.74%) | 2(6.25%) | 4(7.02%) |  |  |
| embA:-12C/T | 5(5.62%) | 2(6.25%) | 3(5.26%) |  |  |
| Others | 20(22.47%) | 4(12.50%) | 16(28.07%) |  |  |
| Eto | 20 | 2 | 16 | 0.0087 | 0.9259 |
| fabG1:-15C/T | 15(75.00%) | 2(100.00%) | 11(68.75%) |  |  |
| Others | 5(25.00%) | 0(0.00%) | 5(31.25%) |  |  |
| **INH** | 124 | 46 | 76 | **8.2277** | **0.04153**† |
| katG:S315T | 79(63.71%) | 37(80.43%) | 42(55.26%) |  |  |
| fabG1:-15C/T | 15(12.10%) | 2(4.35%) | 11(14.47%) |  |  |
| ahpC:-52C/T | 5(4.03%) | 1(2.17%) | 4(5.26%) |  |  |
| Others | 25(20.16%) | 6(13.04%) | 19(25.00%) |  |  |
| LFX/MFX | 79 | 19 | 60 | 6.1710 | 0.1867 |
| gyrA:D94G | 29(36.71%) | 4(21.05%) | 25(41.67%) |  |  |
| gyrA:A90V | 19(24.05%) | 3(15.79%) | 16(2667%) |  |  |
| gyrA:D94N | 10(12.66%) | 4(21.05%) | 6(10.00%) |  |  |
| gyrA:D94A | 7(8.86%) | 3(15.79%) | 4(6.67%) |  |  |
| Others | 14(17.72%) | 5(26.32%) | 9(15.00%) |  |  |
| PZA | 69 | 30 | 37 |  |  |
| pncA:Q141P | 7(10.14%) | 3(10.00%) | 4(10.81%) | 0.0264 | 0.9869 |
| pncA:V131G | 7(10.14%) | 3(10.00%) | 4(10.81%) |  |  |
| Others | 55(79.71%) | 24(80.00%) | 29(78.38%) |  |  |
| RFB | 71 | 27 | 42 | **8.0630** | **0.0447**† |
| rpoB:S450L | 58(81.69%) | 24(88.89%) | 32(76.19%) |  |  |
| rpoB:H445D | 6(8.45%) | 0(0.00%) | 6(14.29%) |  |  |
| rpoB:H445Y | 5(7.04%) | 1(3.70%) | 4(9.52%) |  |  |
| Others | 2(2.82%) | 2(7.41%) | 0(0.00%) |  |  |
| RFP | 107 | 42 | 63 | 10.6110 | 0.1012 |
| rpoB:S450L | 58(54.21%) | 24(57.13%) | 32(50.79%) |  |  |
| rpoB:H445D | 6(5.61%) | 0(0.00%) | 6(9.52%) |  |  |
| rpoB:H445Y | 5(4.67%) | 1(2.38%) | 4(6.35%) |  |  |
| rpoB:S441P | 5(4.67%) | 0(0.00%) | 5(7.94%) |  |  |
| rpoB:D435Y | 5(4.67%) | 3(7.14%) | 2(3.17%) |  |  |
| rpoB:D435V | 5(4.67%) | 3(7.14%) | 2(3.17%) |  |  |
| Others | 23(21.50%) | 11(26.19%) | 12(19.05%) |  |  |
| Sm | 69 | 33 | 34 | 1.2909 | 0.7313 |
| rpsL:K43R | 50(72.46%) | 25(75.76%) | 23(67.65%) |  |  |
| rpsL:K88R | 9(13.04%) | 4(12.12%) | 5(14.71%) |  |  |
| rrs:514A/C | 9(13.04%) | 4(12.12%) | 5(14.71%) |  |  |
| Others | 1(1.45%) | 0(0.00%) | 1(2.94%) |  |  |

Note: §: Total includes new treatment, retreatment and unknown; †: P value is significant; $: Only 244 samples have culture-based DST results; *: Worse represents recurrent, aggravated and dead; #: Under treatment represents first treatment and follow-up treatment; ⁑: MDR/RR excluded Pre-XDR and XDR.
