# Supplementary material for: Whole-genome sequencing reveals transmission pattern and drug resistance of Mycobacterium tuberculosis intra- or inter-hosts
Source: Front Cell Infect Microbiol. 2025 Jan 21;14:1488547. doi: 10.3389/fcimb.2024.1488547 (PMC11790449; doi:10.3389/fcimb.2024.1488547)
Supplement: Supplementary file 10 [file Table9.docx]

Supplementary Table 9. The clinical characteristics of sequenced TB samples infected with MTBC.

| Data | Number | | | X-squared | P value |
| --- | --- | --- | --- | --- | --- |
|  | Total | Non-clustered | Clustered |  |  |
| Lineage | 282 | 84 | 198 | 3.0655 | 0.2159 |
| Lineage1 | 6(2.13%) | 0(0.00%) | 6(3.03%) |  |  |
| Lineage2 | 213(75.53%) | 67(79.76%) | 146(73.74%) |  |  |
| Lineage4 | 63(22.34%) | 17(20.24%) | 46(23.23%) |  |  |
| Lineage2 | 213 | 67 | 146 | 2.6977 | 0.1005 |
| Beijing sublineage | 203(95.31%) | 61(91.04%) | 142(97.26%) |  |  |
| Others | 10(4.69%) | 6(8.96%) | 4(2.74%) |  |  |
| Sample type | 282 | 84 | 198 | 7.6880 | 0.0529 |
| sputum | 123(43.62%) | 42(50.00%) | 81(40.91%) |  |  |
| pleural fluid | 59(20.92%) | 17(20.24%) | 42(21.21%) |  |  |
| BALF | 59(20.92%) | 20(23.81%) | 39(19.70%) |  |  |
| Others | 41(14.54%) | 5(5.95%) | 36(18.18%) |  |  |
| Treatment type | 282 | 84 | 198 | 4.2201 | 0.1212 |
| New | 168(59.57%) | 43(51.19%) | 125(63.13%) |  |  |
| Retreatment | 101(35.82%) | 35(41.67%) | 66(33.33%) |  |  |
| Unknown | 13(4.61%) | 6(7.14%) | 7(3.54%) |  |  |
| Culture-based drug resistance^$^ | 244 | 71 | 173 | 3.0443 | 0.5504 |
| Pre-XDR | 35(14.34%) | 14(19.72%) | 21(12.14%) |  |  |
| MDR^⁑^ | 10(4.10%) | 2(2.82%) | 8(4.62%) |  |  |
| RR^‡^ | 1(0.41%) | 0(0.00%) | 1(0.58%) |  |  |
| DR | 49(20.08%) | 13(18.31%) | 36(20.81%) |  |  |
| DS | 149(61.07%) | 42(59.15%) | 107(61.85%) |  |  |
| Genotype-based drug resistance | 282 | 84 | 198 | 9.5237 | 0.0899 |
| XDR | 5(1.77%) | 2(2.38%) | 3(1.52%) |  |  |
| Pre-XDR | 56(19.86%) | 24(28.57%) | 32(16.16%) |  |  |
| MDR | 28(9.93%) | 6(7.14%) | 22(11.11%) |  |  |
| RR | 8(2.84%) | 1(1.19%) | 7(3.54%) |  |  |
| DR | 39(13.83%) | 7(8.33%) | 32(16.16%) |  |  |
| DS | 146(51.77%) | 44(52.38%) | 102(51.52%) |  |  |

Note: BALF: bronchoalveolar lavage fluid; $: Only 244 samples had culture-based drug susceptibility testing result; ⁑: MDR excluded Pre-XDR and XDR; ‡: RR excluded MDR, Pre-XDR and XDR.
